# Correction to: *BMC Public Health 21:408 & 21:2336*

**DOI:** 10.1186/s12889-024-19053-0

**Published:** 2024-06-10

**Authors:** Hugo Rosado

**Affiliations:** Departamento de Desporto e Saúde, Escola de Saúde e Desenvolvimento Humano, Universidade de Évora, Largo dos Colegiais 2, Évora, Portugal

**Correction to**: ***BMC Public Health*21 (Suppl 2), 408 (2021).** 10.1186/s12889-021-10448-x **and** ***BMC Public Health*21 (Suppl 2), 2336 (2021).**10.1186/s12889-022-13725-5

Following publication of the original articles, the authors brought the following, concerning their use of the ‘MMSE’ instrument to the attention of the journal: We used an unauthorized version of the *Portuguese* MMSE without permission from PAR. PAR have now granted retrospective permission. The MMSE is a copyrighted instrument and may not be used or reproduced in whole or in part, in any form or language, or by any means without written permission of PAR (www.parinc.com).

